# Modelling the Thermal Energy Storage of Cementitious Mortars Made with PCM-Recycled Brick Aggregates

**DOI:** 10.3390/ma13051064

**Published:** 2020-02-27

**Authors:** Christoph Mankel, Antonio Caggiano, Andreas König, Diego Said Schicchi, Mona Nazari Sam, Eddie Koenders

**Affiliations:** 1 Institut für Werkstoffe im Bauwesen, Technische Universität Darmstadt, Franziska-Braun-Straße 3, 64287 Darmstadt, Germany; mankel@wib.tu-darmstadt.de (C.M.); dmsaid@gmail.com (D.S.S.); sam@wib.tu-darmstadt.de (M.N.S.); koenders@wib.tu-darmstadt.de (E.K.); 2 CONICET, LMNI, INTECIN, Facultad de Ingeniería, Universidad de Buenos Aires, C1053 Buenos Aires, Argentina; 3 Department of Prosthodontics and Dental Materials Science, Leipzig University, 04109 Leipzig, Germany; akoenig@uni-leipzig.de

**Keywords:** thermal-energy storage, enthalpy method, apparent calorific capacity method, recycled brick aggregates, meso-scale, PCMs, paraffin waxes, Stefan Problem

## Abstract

This paper reports a numerical approach for modelling the thermal behavior and heat accumulation/liberation of sustainable cementitious composites made with Recycled Brick Aggregates (RBAs) employed as carriers for Phase-Change Materials (PCMs). In the framework of the further development of the fixed grid modelling method, classically employed for solving the well-known Stefan problem, an enthalpy-based approach and an apparent calorific capacity method have been proposed and validated. More specifically, the results of an experimental program, following an advanced incorporation and immobilization technique, developed at the *Institut für Werkstoffe im Bauwesen* for investigating the thermal responses of various combinations of PCM-RBAs, have been considered as the benchmark to calibrate/validate the numerical results. Promising numerical results have been obtained, and temperature simulations showed good agreement with the experimental data of the analyzed mixtures.

## 1. Introduction

The energy demand for heating and cooling the global building stock represents a massive part of the total energy consumption around the world (≈ 40%) [1]. In the EU, it accounts for about half of all energy consumption [2]. To attenuate this number, thermal efficiency of construction and building elements, like walls, roofs, and floors, has become the most important measure to enhance energy savings of the new and existing building stock. Recently, with the introduction of the 2019/2021 EU Buildings Directive, it has been additionally emphasized that new buildings of the EU-Member States have to be designed as “Nearly-Zero-Energy Buildings” from the beginning of 2021 onward [3]. For public non-residential buildings, this obligation should become active in 2019 and will be permanently anchored in future building legislation. Moreover, the German Energy Saving Regulation (EnEV) 2014 has proclaimed 25% stricter requirements for energy savings in their 2016 issue, which affects the set of most important regulations of new and existing German buildings. These regulations define the maximum annual primary energy demand and the maximum permissible loss of transmission heat through the building envelope, on the basis of a reference building. In order to comply with the European targets on energy savings, the German federal government will merge the rules that currently apply in parallel—(i) the Energy Saving Act (EnEG) [4], (ii) the Energy Saving Regulation (EnEV) [5], and (iii) the Renewable Energy Heat Act (EEWärmeG) [6]—into a new Building Energy Act (GEG) [7].

Possible innovative solutions include the potential of materials to embody large amounts of thermal energy, which would stabilize the inner thermal comfort of either residential or non-residential buildings. Particularly, this can be achieved through an effective use of Phase Change Materials (PCMs), which minimizes the additional need of primary energy for heating/cooling [8,9]. Numerous articles on PCMs have been reported in literature and in several fields of applications. The experimental-based research is predominantly addressing the thermo-hydro-chemo-mechanical properties of cementitious materials with PCMs [10,11,12]. In those works, the investigated PCMs are characterized by a melting temperature that varies between 19 °C and 26 °C, which corresponds to a standard temperature range for comfortable living [13]. According to their chemical compositions, PCMs can be categorized as organic, inorganic and eutectics [14], while according to their phase transition mode as liquid–gas, solid–gas, solid–liquid, and solid–solid. Solid–liquid is the most preferred mode for energy and building applications [15,16].

A large number of review studies are already available in literature, which are very helpful for understanding the State of the Art (SoA) of the current research, and also to evaluate the potential directions for future investigations. In a very recent review paper, performed at TU-Darmstadt [17], the authors reviewed the various numerical tools that are used for modelling phase change phenomena in problems of materials science and engineering. The available modelling strategies for PCM-based cementitious composites, at different scale levels (micro-, meso-, macro-, and multiscale [18]), have been deeply explored, evaluated and discussed.

Numerical tools and theoretical approaches, available in scientific literature for modelling Thermal Energy Storage (TES) and heat accumulation/liberation of PCM-based applications, deal with solving the so-called Stefan problem [19] or an extended version of it. The Stefan problem assumes the existence of two different domains, one representing the solid phase (*Ω_S_*) and another representing the liquid phase (*Ω_L_*). These are separated by a sharp front, identified as a moving interface (*Γ*), which is the location where the phase transformation takes place. Such sharp surface front is characterized by a temperature field that equals the melting temperature *T_m_*. Several authors, under simplified assumptions (like 1D conduction-only heat problem in both the solid and liquid phase), have proposed analytical solutions for this problem [20]. While, more complex models can be taken into account for further evaluation of the classical Stefan problem: e.g., by introducing a so-called “mushy zone” representing non-isothermal conditions and dealing with a finite thickness of this moving interface zone, where the liquid and solid phases may coexist [21]. The solution method of the Stefan problem and/or its extended formulation, can generally be subdivided into three different categories [22]: (i) the fixed grid method, where the grid of spatial nodes, used for discretizing the problem, remains fixed during time, thus the phase change is traced through auxiliary constitutive formulations and state functions; (ii) the deformed grid method, where the nodes forming the grid may move to explicitly follow the moving sharp front that occurs during the melting and/or solidification; (iii) and hybrid methods, which are a combination of (i) and (ii). Most classical examples are those based on a further development of the fixed grid method through following the enthalpy-based approach (EA) [23], which mainly boils down into two alternative solutions: (i) the apparent calorific capacity method (ACCM) [24] and (ii) the heat source method (HSM) [25].

In this context, the present study investigated the TES of mortars made of Recycled Brick Aggregates containing PCMs. The main scope was to assess a numerical procedure for simulating the effects of paraffin waxes on the thermal energy responses of mortars produced with different types and amounts of PCM-RBAs. The results of a wide series of thermal tests [26] (DSC measurements, thermal conductivity and special spherical tests, labelled DKK—*Dynamische Kugel Kalorimetrie*), carried out on PCM, aggregates, plain mortars, and PCM-RBA mortars are summarized. The aforementioned experimental results are employed in an inverse identification procedure for unveiling the key parameters that drive the TES of the mixtures.

Following this short introductive literature review, the paper is structured as follows. Section 2 addresses the enthalpy-based model and the ACCM resolution of it, employed for simulating the phase transformation phenomena of the PCM-RBA mortars. Experimental results, outlined in terms of TES results, are shown in Section 3. Section 4 outlines the spherical-based solution and numerical implementation of the model procedure described in Section 2. In Section 5, numerical results and comparisons against the experimental data are reported and discussed to demonstrate the soundness and capability of the numerical procedure. Concluding remarks and future research outlook are addressed in Section 6.

## 2. The Enthalpy-Based Model and Apparent Calorific Capacity

This section reports the enthalpy-based model that will be employed for predicting the phase transformation phenomena of PCM-RBA mortars.

### 2.1. Basic Principles: Thermodynamics and Thermal Energy Storages

The enthalpy *H* of a thermodynamic system can be defined as

(1) H=U+pV

with *U* the internal energy, *p* the pressure and *V* the volume of the system.

Then, by introducing the first law of thermodynamics, for closed systems and an infinitesimal process, the following equation can be stated as

(2) dU=δQ−δW

i.e., the variation of the internal system energy (*dU*) is equal to an infinitesimal amount of heat added (δQ) minus an infinitesimal amount of work performed (δW).

Under the hypothesis that only *p* and *V* spend work (quasi-static process), δW=p dV.

It follows that

(3) dU=δQ−p dV.

Now, by evaluating *dH* from Equation (1) and using Equation (3), the following equation will be achieved:

(4) dH=dU+d(pV)=dU+dpV+pdV=δQ−pdV+dpV+pdV=δQ+dpV.

For a constant pressure, which represents most cases of PCM-concrete systems of construction and building applications, it can be assumed that a small variation of enthalpy is equal to a small amount of heat added:

(5) dH=δQ.

### 2.2. Enthalpy Description and Apparent Calorific Capacity Method (ACCM)

In accordance with the fixed grid modelling method and with its discretization through the ACCM approach (see reference [17]), the classical equation for describing a heat conduction problem can written as follows:

(6) $$\frac{\partial Q}{\partial t}=\nabla .\left(\lambda \nabla T\right)+{\dot{q}}_{v}\;\forall \;x\in \Omega$$

where *Q* is the heat of the system, *t* the time, λ=λ(T,x) the thermal conductivity of the material, which depends on the temperature *T* and position vector x (of the considered body Ω), ${\dot{q}}_{v}$ is the possible source term, while ∇. and ∇ are the divergence and gradient tensorial operators.

Substituting Equation (5) into Equation (6) leads to the following equation:

(7) $$\frac{\partial H}{\partial t}=\nabla .\left(\lambda \nabla T\right)+{\dot{q}}_{v}\;\forall \;x\in \Omega$$

which is the mostly adopted equation for solving phase changes in simulations for construction and building applications, and it is known in literature as the enthalpy-based method.

Applying the chain rule to $\frac{\partial H}{\partial t}$ of Equation (7),

(8) $$\frac{\partial H}{\partial t}=\frac{\partial H}{\partial T}\frac{\partial T}{\partial t}$$

and by introducing the concept of the Apparent Calorific Capacity Method (ACCM), the following temperature-dependent (apparent of effective) heat capacity expression can be written [17]:

(9) $$\frac{\partial H}{\partial T}=\rho C_{eff}\left(T\right)$$

Thus, Equation (7) modifies into the following heat transfer equation.

(10) $$\rho C_{eff}\left(T\right)\frac{dT}{dt}=\nabla .\left(\lambda \nabla T\right)+{\dot{q}}_{v}\;\forall \;x\in \Omega$$

Finally, the following initial condition (IC) and boundary conditions (BCs) complete the description of the phase change problem:

(11) $$\mathrm{IC}:\;\;T\left(x,t=0\right)=T_{0}\left(x\right)\;\forall \;x\in \Omega$$

(12) $$\mathrm{BCs}:\;\;\begin{array}{ll} T\left(x,t\right)=T_{D} & \;\forall \;x\in \Gamma_{T}\\ \left(\lambda \nabla T\right)\cdot n=q & \;\forall \;x\in \Gamma_{q}\\ \left(\lambda \nabla T\right)\cdot n=h\left(T_{env}-T\right) & \;\forall \;x\in \Gamma_{c}\\ \left(\lambda \nabla T\right)\cdot n=\kappa \left(T_{rad}-T\right) & \;\forall \;x\in \Gamma_{r} \end{array}$$

where $\Gamma_{T},\;\Gamma_{q},\;\Gamma_{c}\;\mathrm{and}\;\Gamma_{r}$ are the essential and natural (flux, convection and radiation) boundary conditions of the domain Ω. *T_D_* is the specified temperature imposed at the essential boundary, *q* the heat flux, *h* the heat convection and *κ* the radiation coefficients, respectively, $T_{env}$ the environmental temperature, and, finally, *T_rad_* the external radiation source temperature.

## 3. Overview of the Experimental Test Data

This section reports the results of an experimental program performed for characterizing the thermal-energy response of PCM-RBA mortar mixtures and their components. The experimental data are used for validation of the numerical simulations discussed in Section 5.

Six mixtures were considered, having a w/c ratio of 0.5 and various amounts of PCM-RBA volume fractions (Table 1). All mixtures were prepared according to EN 196-1 [27]. Recycled bricks (labelled “SB”) and high porosity Poroton® fired-clay blocks (labelled “PB”) have been considered [28]. These building materials, processed in the form of medium/coarse aggregates and provided by a local company (SHW GmbH, Messel, Germany), were used as carriers (containers) for storing a predefined volume of PCM.

RT 25 HC [29] paraffin waxes were used as PCM. They are characterized by a high crystallinity and possess an excellent heat store capacity during phase changes, from solid to liquid and vice versa. The thermo-physical properties of the paraffin wax, considered in this research, are a melting temperature of 25 °C, a storage capacity of approximately 230 kJ/kg, a latent heat capacity of almost 200 kJ/kg, thermal conductivity (in both phases) of 0.20 W/m × K, and densities of 880 (liquid)/770 (solid) kg/m³. The PCM-RBAs were thus produced following an advanced encapsulation technique, proposed and patented by the *Institut für Werkstoffe im Bauwesen* of TU-Darmstadt (Darmstadt, Germany) [30].

The mixture names (labels) in Table 1 aim at providing the key information on the amount of PCM, filling the RBA’s open porosity (expressed in volume fraction of the open capillary pore space), and the type of RBAs considered in the mixture. For example, the label ‘‘REF-SB’’ refers to the reference mixture (without PCM) using SB type of bricks; or “SB-65” indicates a mixture using SB-bricks and a filling degree of PCM of 65 V.-% of the total SB-RBA open capillary porosity. The complete description (materials, methods, results and discussion) of the experimental campaign is available in Mankel et al. [28].

### 3.1. DSC Measurements: Aggregates, Paste, and PCMs

**Figure 1:**
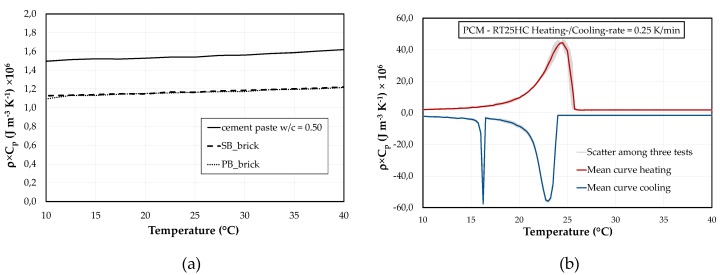
ρ × C_p_ results of DSC measurements of (**a**) w/c = 0.50 cement pastes, SB and PB obtained with a heating/cooling rate of 10 K × min^−1^ and (**b**) Paraffin RT25HC with a heating/cooling rate of 0.25 K × min^−1^.

Differential Scanning Calorimetry (DSC) tests were performed for each component used in the investigated PCM-RBA mortar mixtures. Their heat storage capacity has been expressed in terms of bulk density times the specific heat capacity, i.e. ρ × C_p_. Three samples per each component were analyzed and the mean value for each of them was presented in this section. Particularly, the DSC thermograms under either heating or cooling, for the cement paste with a w/c of 0.50 and for both SB- and PB-RBAs, have been shown in Figure 1a. They were done within the temperature range of 10 °C to 40 °C and using a heating/cooling rate of 10 K × min^−1^. From these results, it can be observed that the sensible behavior of both RBAs is almost similar, whereby a slightly higher sensible heat storage capacity can be detected for the cement paste.

In Figure 1b, the DSC results of the used paraffin wax (Rubitherm® RT25HC, Rubitherm Technologies GmbH, Berlin, Germany) is shown. The test procedure was conducted in accordance with the IEA DSC 4229 PCM Standard [31] to determine the final heating/cooling rate for the considered dynamic DSC measurements. The adopted heating/cooling rate was 0.25 K × min^−1^ on the final measured results. This value represented a compromise between accuracy of the results and acceptable mitigation of heating rate measurements, fulfilling the IEA DSC 4229 requirements [31].

DSC curves of the considered PCM, in Figure 1b, shows a sensible heat storage character of the material in those temperature ranges, far from the phase change responses (e.g., in the solid and liquid stages), and shows a pronounced latent peak in the region close to the temperature where the phase change occurs (T_m_ = 24.5 °C for heating and T_m_ = 22.95 °C for cooling).

### 3.2. Thermal Conductivity of the PCM-RBA Mortars

**Figure 2:**
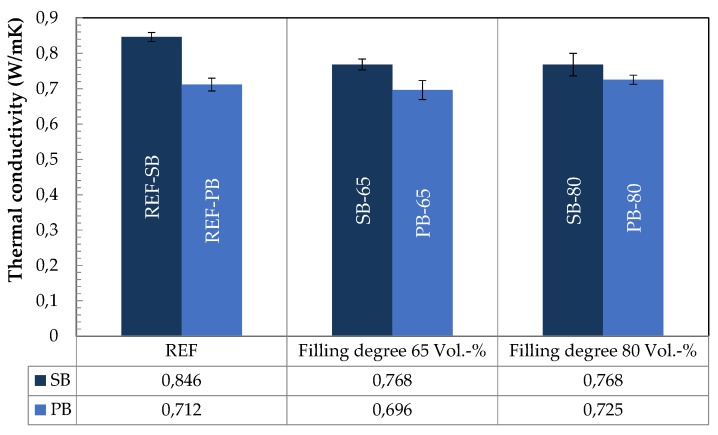
Thermal conductivity of the SB- and PB-RBA mortars, with and without PCMs.

The thermal conductivity of PCM-RBA mortar mixtures was determined using the Hot-Disk transient plane source method [28]. For this aim, three samples of 150 mm × 150 mm × 80 mm cuboids were tested. The measurements were done with a 9.9 mm diameter sensor, in three different specimen sides. Steady-state conditions with a temperature of 20 °C were considered. In Figure 2, it can be observed that all mixtures have quite comparable thermal conductivities, which range between 0.696 (min. value) and 0.846 (max. value) W/mK. As a general trend, it can be observed that the mixtures with PB RBAs deal with slightly lower conductivities than the SB RBA ones. This can be attributed to the higher porosity of the SB RBAs, which affect the overall conductivity of the composites. It could also be observed that for higher PCM contents, the thermal conductivity is lower.

Moreover, it can be also observed that the thermal conductivities are only slightly affected by the different PCM volume fractions. A little increase of the thermal conductivity was measured for those mixtures with a higher pore filling ratio (i.e. PB-80 in comparison with PB-65). This could be the result of the allocation of paraffin wax into the capillary pore space of the composites, which leads to a slight increase in heat conductivity of the PCM-RBAs since the considered PCMs are more conductive than air. However, it can finally be concluded that immobilizing PCMs into the RBA porous structure does basically not (e.g. SB-80 vs. SB-65), or only slightly (see, PB-80 vs. PB-65), modify the overall thermal conductivity of RBA mortars.

### 3.3. Thermal-Energy Storage DKK Tests in Spherical Samples

Spherical-shaped specimens were used to monitor the time-dependent temperature evolution of the PCM-RBA mortars. The adopted and patented non-conventional testing technique (namely *Dynamische Kugel Kalorimetrie*), DKK [26]) was followed by the authors for the TES identification of the composite materials under investigation. For each considered mixture, three spherical samples were produced, and two thermocouples were positioned in the center of each sphere and at its outer surface, respectively. Heating tests were done by using an isothermal conditioned oven with a fixed temperature of 49 °C. Cooling tests were done with a climatic chamber fixing the temperature at ca. 9 °C.

**Figure 3:**
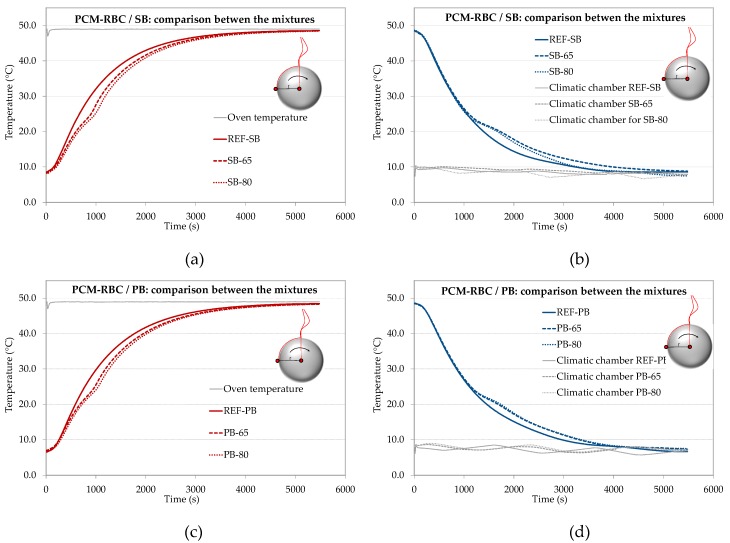
Temperature evolutions of the DKK tests: (**a**) heating and (**b**) cooling of PCM-RBA mortars with SB bricks, (**c**) heating and (**d**) cooling of PCM-RBA mortars with PB bricks. These results represent the average of three measurements in the center of the spherical samples.

The graphs of Figure 3 show the average results (from three independent spherical specimens) of the measured temperature evolution, in the center of the spherical samples, versus time. Heating and cooling results are plotted for both PCM SB-brick and PB-brick mixtures. It can be seen that, for the mortar mixtures casted with either SB or PB bricks, a delay of the temperature development takes place when PCM-RBAs, with PCM filling degrees of 65 V.-% and 80 V.-%, are analyzed. The presence of PCM and their melting/solidification behavior actually shifts the temperature curve into the right/down direction for heating response (Figure 3a,c) and up/left for cooling (Figure 3b,d). Particularly, a quasi-horizontal plateau of the temperature evolution data can be appreciated during both temperature rise (heating) or temperature decrease (cooling), i.e. in the range between 21 and 26 °C the PCM and vice versa. By taking into consideration the effect of PCM volume fractions, it appeared that almost no thermal differences between the considered RBAs and PCM additions, i.e., 65 and 80%, exist. Comparison of the temperature evolutions SB-65 with SB-80 or PB-65 with PB-80 show an almost equal response. For each mixture and/or sample, the complete phase change of the paraffin was always fully occurring, meaning that all PCMs were in their final state at the end of each heating or cooling test. This is also shown in Figure 3 where the center of the spheres at the end of each cooling test has a temperature less than 10 °C and under heating close to 50 °C, while the melting points range between 21–26 °C.

For a thorough discussion on the present experimental data, with emphasis on the DSC analyses, conductivity measurements and DKK tests, reference is made to Mankel et al. 2019 [28].

## 4. Numerical Implementation and Spherical-based Solution

### 4.1. D Spherical-Based Solution for the ACCM

Equation (10) is now transferred into spherical coordinates for predicting the thermal energy storage behavior and temperature evolution in the tested specimens presented in Section 3.3.

From this, the following relation can be derived:

(13) $$\rho C_{eff}\left(T\right)\frac{dT}{dt}=\frac{1}{r^{2}}\frac{\partial }{\partial r}\left(\lambda r^{2}\frac{\partial T}{\partial r}\right)+\frac{1}{r^{2}\sin^{2}\theta }\frac{\partial }{\partial \phi }\left(\lambda \frac{\partial T}{\partial \phi }\right)+\frac{1}{r^{2}\sin\theta }\frac{\partial }{\partial \theta }\left(\lambda \sin\theta \frac{\partial T}{\partial \theta }\right)+{\dot{q}}_{v}$$

where (r,θ,ϕ) are the radial distance and polar and azimuthal angles. Making use of the spherical symmetry, the previous relationship can be significantly simplified as follows:

(14) $$\rho C_{eff}\left(T\right)\frac{dT}{dt}=\frac{1}{r^{2}}\frac{\partial }{\partial r}\left(\lambda r^{2}\frac{\partial T}{\partial r}\right)+{\dot{q}}_{v}.$$

### 4.2. Schematization and Discretization

The ACCM model, under the assumptions of spherical geometry and symmetry, was solved by means of the finite difference method. The heat-diffusion through the PCM-RBA mortar systems was calculated by solving the differential equation previously described in Equation (14), having a 1D spherical-based hypothesis and by adopting heat convection (Robin) boundary conditions for describing the environmental surface conditions of either a furnace or a climate chamber.

**Figure 4:**
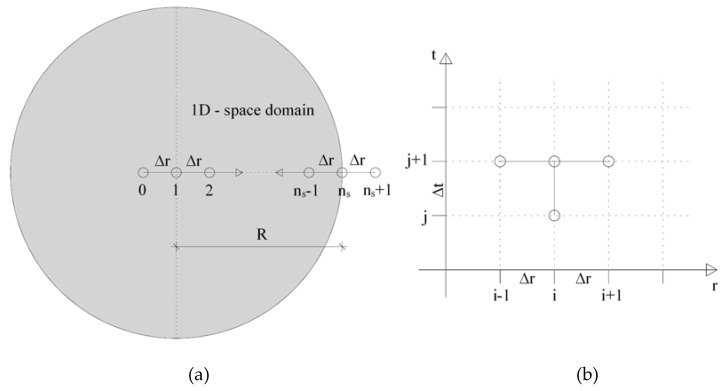
(**a**) Finite difference (FD) space discretization scheme of a 1D-heat transfer in spherical specimens; (**b**) FD molecule for implicit time using the Backward Euler Method and the Space Central Method.

Thus, the boundary condition for the sample core at node “1” (Figure 4) was adiabatic, meaning that the heat flux, *q*_1_, is null.

(15) $$\;\mathrm{Sample}\;\mathrm{core}:\;\;q_{1}=-\lambda {\left(\frac{\partial T}{\partial r}\right)|}_{r=0}=0$$

while the Robin boundary condition is applied at the outer surface (node “*n_s_*”)

(16) $$\text{Ambient condition}:\;\;q_{n_{s}}=-\lambda {\left(\frac{\partial T}{\partial r}\right)|}_{r=R}=h\left(T_{f}-T_{r}\right)$$

where *T_f_* is the ambient temperature (fixed by the oven or climatic chamber), *T_r_* is the surface temperature at *r = R*, while *h* is the heat transfer coefficient.

In this context, the Finite Difference (FD) space domain was discretized into *n_s_* spaces (namely Finite Differences), leading to *n_s_* + 1 nodes and *n_t_* time discretization steps. Furthermore, a fully implicit Euler Method for the transient problem was applied.

The differential equation shown in Equation (14), within a space domain with a length of R between the node 1 to *n_s_* (see Figure 4a), can be solved as shown in the following scheme:

(17) $$\rho C_{eff}\left(T\right)\frac{dT}{dt}=\frac{2\;\lambda }{r}\frac{\partial T}{\partial r}+\lambda \frac{\partial^{2}T}{\partial r^{2}}\;\mathrm{with}\;r=\left(i-1\right)\Delta r$$

By using the implicit backward Euler Method for time (*j*) and central for space (*i*) (see Figure 4b), Equation (17) can be discretized to

(18) $$\rho C_{eff}\left(T_{i}^{j+1}\right)\frac{T_{i}^{j+1}-T_{i}^{j}}{\Delta t}=\lambda \frac{T_{i+1}^{j+1}-T_{i-1}^{j+1}}{\left(i-1\right)\Delta r^{2}}+\lambda \frac{T_{i-1}^{j+1}+2T_{i}^{j+1}+T_{i+1}^{j+1}}{\Delta r^{2}}.$$

The boundary condition, for the sample core at *r = 0* (*i* = 1), has been further developed. The right-hand side of Equation (17) can be simplified as follows [32]

(19) $$\frac{\lim}{r\to 0}\left(\frac{2\;\lambda }{r}\frac{\partial T}{\partial r}+\lambda \frac{\partial^{2}T}{\partial r^{2}}\right)=3\lambda \frac{\partial^{2}T}{\partial r^{2}}$$

Then, by adopting the central difference approximation of the adiabatic boundary condition expressed of Equation (15) and by using one ghost node (namely node “0”), the following expression can be stated:

(20) $$-\lambda {\left(\frac{\partial T}{\partial r}\right)|}_{r=0}=-\lambda \frac{T_{2}^{j+1}-T_{0}^{j+1}}{2\Delta r}=0$$

It follows that $T_{2}^{j+1}=T_{0}^{j+1}$ and combining it into Equations (18) and (19), it can be easily achieved the following expression of the adiabatic boundary condition:

(21) $$\rho C_{eff}\left(T_{1}^{j+1}\right)\frac{T_{1}^{j+1}-T_{1}^{j}}{\Delta t}=6\lambda \frac{T_{2}^{j+1}+T_{1}^{j+1}}{\Delta r^{2}}$$

For the implementation of the Robin boundary condition at the outer surface of the specimens, e.g. node *r = R* (*i = n*_s_), Equation (15) can be discretized in the following way:

(22) $$-\lambda \frac{T_{n_{s}-1}^{j+1}-T_{n_{s}+1}^{j+1}}{2\Delta r}=h\left(T_{f}^{j+1}-T_{r}^{j+1}\right)$$

In Equation (22), the temperature at the ghost node *r = R* + 1 (*i = n_s_* + 1) is known, $T_{n_{s}+1}^{j+1}=T_{f}$, thus the following expression can be rewritten:

(23) $$T_{n_{s}+1}^{j+1}=\frac{2\;\Delta r\;h}{\lambda }\left(T_{n_{s}}^{j+1}-T_{f}^{j+1}\right)+T_{n_{s}-1}^{j+1}$$

Thus, Equation (16) can be easily written as

(24) $$\begin{array}{l} \rho C_{eff}\left(T_{n_{s}}^{j+1}\right)\frac{T_{n_{s}}^{j+1}-T_{n_{s}}^{j}}{\Delta t}=\lambda \frac{\left(\frac{2\;\Delta r\;h}{\lambda }\left(T_{n_{s}}^{j+1}-T_{f}^{j+1}\right)+T_{n_{s}-1}^{j+1}\right)-T_{n_{s}-1}^{j+1}}{\left(i-1\right)\Delta r^{2}}+\\ \;\lambda \frac{T_{n_{s}-1}^{j+1}+2T_{n_{s}}^{j+1}\left(\frac{2\;\Delta r\;h}{\lambda }\left(T_{n_{s}}^{j+1}-T_{f}^{j+1}\right)+T_{n_{s}-1}^{j+1}\right)}{\Delta r^{2}} \end{array}$$

and after some mathematical elaborations, the following expression can be achieved for the Robin natural boundary condition:

(25) $$\rho C_{eff}\left(T_{n_{s}}^{j+1}\right)\frac{T_{n_{s}}^{j+1}-T_{n_{s}}^{j}}{\Delta t}=2\left[h\left(\frac{T_{n_{s}}^{j+1}-T_{f}^{j+1}}{\left(i-1\right)\Delta r}+\frac{T_{n_{s}}^{j+1}-T_{f}^{j+1}}{\Delta r}\right)+\lambda \left(\frac{T_{n_{s}-1}^{j+1}+T_{n_{s}}^{j+1}}{\Delta r^{2}}\right)\right]$$

## 5. Numerical Results and Comparisons

This section reports the description of the numerical results and shows their comparisons against the experimental data, which has been briefly outlined in Section 3. The numerical simulations are based on the assumption that the PCM-RBA mortars can be considered as a continuum and homogenous media. In this context, homogenized (meso-scale based) parameters were considered in the selection of the input data for the numerical prediction.

### 5.1. Homogenized Macroscopic C_eff_ Model for the PCM-RBA Mixtures

A homogenization technique was employed for evaluating the effective specific heat capacity *C_eff_* of the PCM-RBA mortars. It is based on the mixture theory by using the volume percentages of each individual component such as RBAs, cement paste and PCMs. More precisely, the model smears out the specific heat capacity of the RBA *C_RBA_(T)*, cement paste *C_paste_(T)* and the apparent specific heat capacity of the PCM *C_app,PCM_(T)* through adopting the volume fraction of each component as the smeared out (weighting) factor *χ*.

The specific heat capacities of each component were experimentally determined with DSC measurements (see Section 3.1) and are shortly summarized in Table 2.

RBAs and cement pastes have only the sensible heat storage part (Figure 1a), while the PCMs have an apparent specific heat capacity *C_app,PCM_(T)* that incorporates the additional latent behavior within the temperature range of the phase change (during melting and solidification, as shown in Figure 1b).

The evaluation of the specific heat capacities was determined in two consecutive steps. First, at aggregate level, where the PCM-RBAs were considered as lumped components of RBAs (*C_RBA_(T)*) plus PCM (*C_app,PCM_(T)*) and weighting their volume fractions χ to achieve the smeared *C_eff,PCM-RBA_(T):*

(26) $$\text{PCM-RBAs}:\;\;\;C_{eff,PCM-RBA}(T)\;=\chi_{RBA}\times C_{RBA}(T)\;+\chi_{PCM}\times C_{app,PCM}(T)$$

where *χ_RBA_* and *χ_PCM_*are the volume fraction of the recycled bricks and the filled PCMs, respectively.

Then, at a composite level (i.e., PCM-RBA mortar) a homogenized overall system of the effective specific heat capacity, *C_eff_*, was determined by weighting the heat capacities *C_eff,PCM-RBA_(θ)*, evaluated through Equation (26), and *C_paste_(θ)* of the individual material components by their volume fractions *ψ:*

(27) $$\text{PCM-RBA mixtures}:\;C_{eff}(T)\;=\psi_{paste}\times C_{paste}(T)\;+\psi_{PCM-RBA}\times C_{eff,PCM-RBA}(T)$$

**Figure 5:**
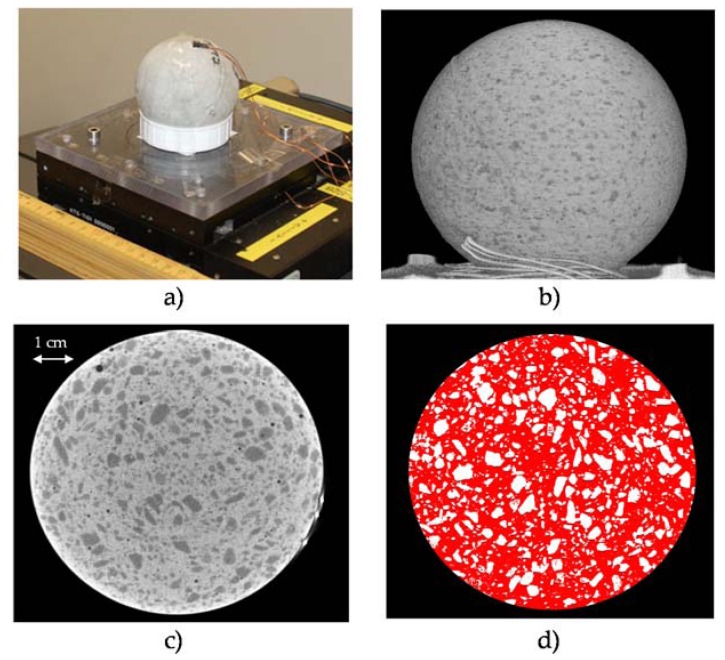
μ3D-XCT-scans setup (**a**,**b**) and slices cross including image-segmentation (**c**,**d**) of the spherical specimens.

**Figure 6:**
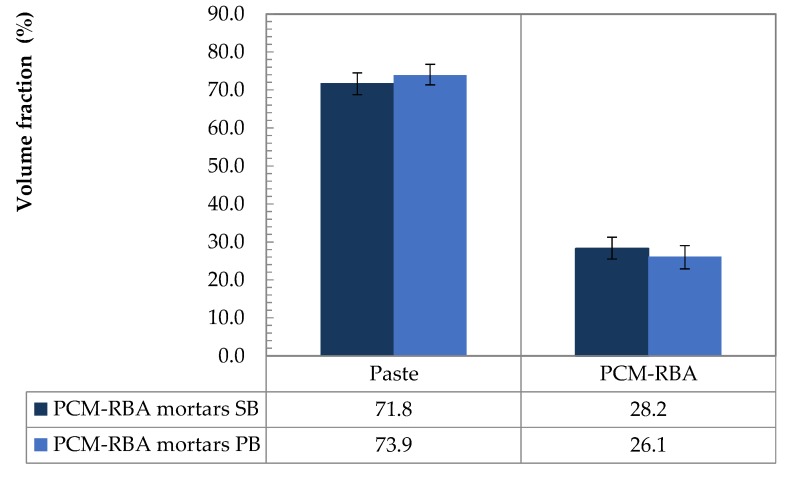
Volume fractions of paste and PCM-RBA determined by image-analysis.

The exact volume fraction ratio between PCM-RBAs and cement paste, of the investigated mixtures, were investigated by performing μ3D-XCT-scans [33] of the spherical specimens. Nine 2D-slices were extracted from each 3D body of the scanned spherical specimens (Figure 5) and mean volume fractions of PCM-RBAs and cement paste were accurately determined by image analyses. An average area ratio was evaluated for each slice by applying a recoloring of the surface area through white and red pictures (Figure 5). The results of these analyses are shown in Figure 6 where the volume fractions of paste and PCM-RBA were determined in both PCM.RBA mortars SB and PB.

The effective specific heat capacity of the composite systems, *C_eff_(T)*, were thus modelled using Equations (26) and (27) by taking into account the determined volume fractions between cement paste (including air voids) and PCM-RBAs (shown in Figure 6) and considering the specific heat capacities of each component as summarized in Table 2.

**Figure 7:**
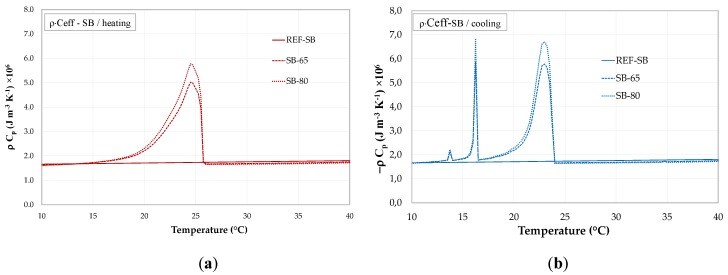
ρ × Ceff of PCM-RBA mortar systems with SB bricks: (**a**) heating and (**b**) cooling.

**Figure 8:**
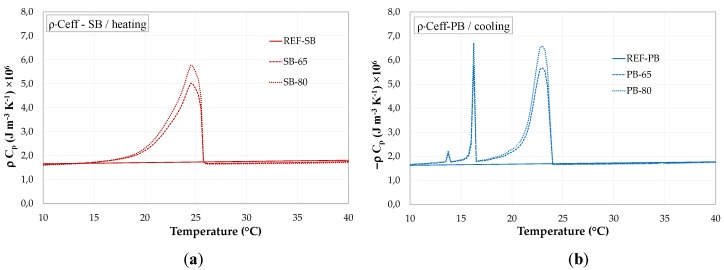
ρ × Ceff of PCM-RBA mortar systems with PB bricks: (**a**) heating and (**b**) cooling.

Figure 7 and Figure 8 shows the afore described *C_eff_(T)* further multiplied by the bulk density *ρ* of the PCM-RBA mortar systems (*ρ* = 1829.6 kg/m³ for REF-SB, 1710.37 kg/m³ for SB-65, 1671.6 kg/m³ for SB-80, 1853.5 kg/m³ for REF-PB, 1767.25 kg/m³ for PB-65 and 1739.8 kg/m³ for PB-80).

### 5.2. Numerical Prediction and Comparison

By implementing the ACCM procedure described in Section 2 and Section 4, temperature evolutions were simulated and compared with the experimental data reported in Section 3. More in detail, the spherical samples made of RBA mortars (with and without PCMs) were simulated with the proposed heat flow model. The input values were obtained from the conducted experimental measurements, as well as from the *C_eff_(T)* curves described in Section 5.1. All input parameters employed in the aforementioned simulations are summarized in Table 3 and Table 4.

The thermal conductivities were assumed as temperature-independent and measured through Hot-Disk tests, as outlined in Section 3.2. Then, the effective specific heat capacity *C_eff_(T)* was modelled by using the homogenized model as described before in this section. The number of FD space discretization was chosen 100 while the number of time steps selected was 1000, in all simulations. Moreover, the calibrated Robin heat transfer coefficient (h), representing the heat transfer conditioning coefficient was the same for each mixture and reported in Table 3 and Table 4.

**Figure 9:**
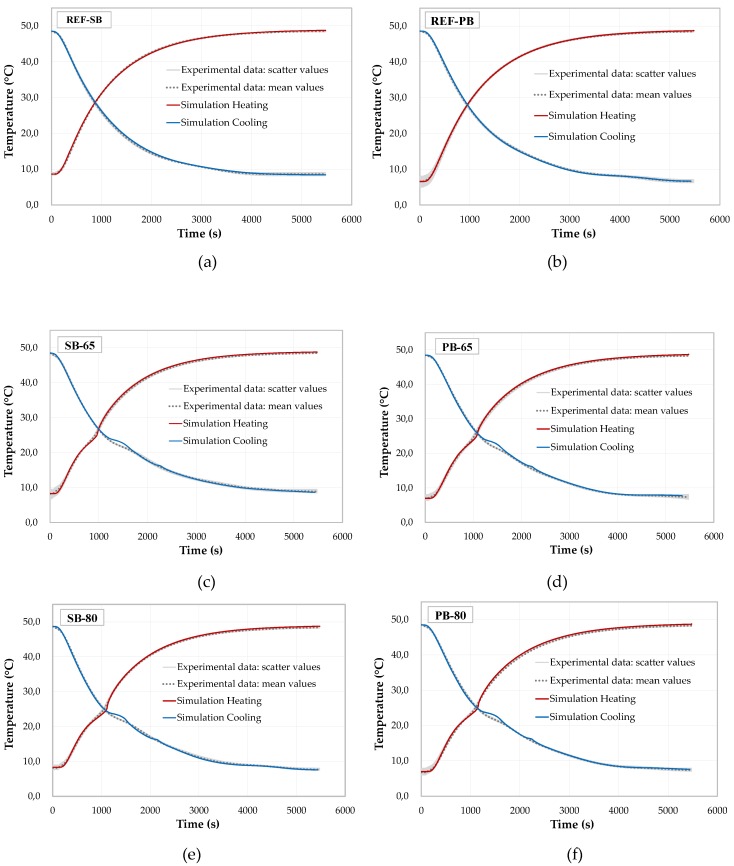
Experimental “DKK” vs. numerical results: (**a**,**c**,**e**) SB brick type mixtures and (**b**,**d**,**f**) PB brick type mixtures.

The numerical simulations, based on the input parameters above mentioned, have been compared against the experimental data of Section 3.3. In Figure 9, the six graphs show the temperature evolutions for the control mixtures (Figure 9a,b), and for that one having PCM: i.e., SB-65 (Figure 9c), SB-80 (Figure 9e), PB-65 (Figure 9d), and PB-80 (Figure 9f). The experimental scatter of the result data for each mixture have been also plotted in grey.

It can be observed that the modeling approach was able to simulate the experimental temperature evolutions very accurately. In particular, the simulations of the reference mixtures represented in Figure 9a,b show a very good agreement with the experimental results. Then, the simulations of the mixtures TES enhanced with PCM also show good comparisons and trends as the experimental values. A slightly overestimated latent effect can be observed, which results in a slightly amplified shoulder in the temperature evolution (see Figure 9c–f). A reason for this effect can be attributed to micro- and/or meso-structural effect, which can influence the effective thermal conductivity and the melting/solidification activations of the integrated PCMs. This could lead to a slight deviation of the latent effects.

In this context, it may be important to remark that almost all input parameters were chosen from the characterizations of the thermal tests as well as from the homogenized C_eff_ model, which is actually represented by the experimentally determined heat capacities of the individual material components weighted by their volume fractions (see Section 5.1). Thus, with this set of input parameters, a sound numerical prediction, for all six PCM-RBA mortar systems, could be achieved, without the need of applying re-calibration and/or optimizations. The results also show that, with a unique heat transfer coefficient, described as the *h* (Robin) parameter, the simulations for the reference PCM-RBA mortar systems, REF-SB and REF-PB, are almost in perfect agreement with the corresponding experiments. This supports the assumption that both the spherical symmetry and the hypothesis that the composite PCM-RBA mortar systems could be considered by a homogeneous medium, were both effectively correct.

## 6. Conclusions

Based on the results shown in this paper, the following conclusions can be drawn:

- An enthalpy-based model, formulated for spherical coordinates and symmetry, was proposed for predicting the thermal energy storage in the tested PCM-RBA specimens.
- Thermal measurements, obtained from dynamic DSC and steady-state Hot Disk tests, were employed for calibrating the model of the numerical activities.
- A mixture theory, based on volume fractions deduced from 3D micro μX-ray computer tomography measurements, was used for generating the resulting meso-composite thermal parameters adopted in the numerical analysis. Particularly the *C_eff_(T)* curves have been based on this approach.
- The numerical simulations for the temperature evolution, compared with the experimental DKK results, showed accurate and consistent agreement. It may be important to highlight that these numerical results were just based on input parameters obtained from the experimental characterizations of the mortar components.
- No fitting adjustments, re-calibrations, or numerical adaptions were necessary for reaching good agreement between numerical to experimental data. This can confirm that on the one hand the experimental activities were performed in an accurate way, and on the other hand, that the numerical assumptions and procedures are very accurate to model TES responses in cementitious materials.

Future numerical developments, which follow this research, will include micro-to-meso scale analysis taking into considerations local effects like inclusions (PCM-RBAs), air bubbles, porosity, and interface effects. These further steps will lead to optimizing the “best” recipe for achieving the most performing energy-saving and sustainable cementitious composite.

## Figures and Tables

**Table 1:** Overview of the six PCM-RBA mortars.

Labels	REF-SB	SB-65	SB-80	REF-PB	PB-65	PB-80
Cement [kg/m^3^]	701.5	694.2	692.9	700.2	691.7	691.7
Water [kg/m^3^]	350.8	347.1	346.5	350.1	345.9	345.9
PCM-RBA [kg/m^3^]	655.2	719.3	734.0	664.0	728.9	743.9
Air cont. [V.-%]	2.3	2.9	3.0	2.4	3.1	3.1
w/c ratio [-]	0.50					

**Table 2:** Overview of the six PCM-RBA mortars.

Cement Paste	RBA	PCM	
*C_paste_* Solid phase	*C_RBA_* Solid phase	C_app,PCM_	Liquid phase Phase change Solid phase
Figure 1a	Figure 1a	Figure 1b	

**Table 3:** Overview of the numerical parameters assumed for the SB mixtures.

Numerical Parameters	REF-SB	SB-65	SB-80
*ρ C_eff_* (J cm^−3^ K^−1^)	Figure 7		
*λ* (W/m K) (Figure 2)	0.846	0.768	0.768
*h* (W m^−2^ K^−1^)	25.0		

**Table 4:** Overview of the numerical parameters assumed for the PB mixtures.

Numerical Parameters	REF-PB	PB-65	PB-80
*ρ C_eff_* (J cm^−3^ K^−1^)	Figure 8		
*λ* (W/m K) (Figure 2)	0.712	0.696	0.725
*h* (W m^−2^ K^−1^)	25.0		
